# The Influence of Light Quality, Circadian Rhythm, and Photoperiod on the CBF-Mediated Freezing Tolerance

**DOI:** 10.3390/ijms140611527

**Published:** 2013-05-30

**Authors:** Punyakishore Maibam, Ganesh M. Nawkar, Joung Hun Park, Vaidurya Pratap Sahi, Sang Yeol Lee, Chang Ho Kang

**Affiliations:** Division of Applied Life Sciences (BK21 program), Gyeongsang National University, Jinju 660-701, Korea; E-Mails: punya.maibam@gmail.com (P.M.); ganeshtnau@gmail.com (G.M.N.); jazzc@nate.com (J.H.P.); vpsahi@hotmail.com (V.P.S.)

**Keywords:** CBF regulon, cold acclimation, circadian clock

## Abstract

Low temperature adversely affects crop yields by restraining plant growth and productivity. Most temperate plants have the potential to increase their freezing tolerance upon exposure to low but nonfreezing temperatures, a process known as cold acclimation. Various physiological, molecular, and metabolic changes occur during cold acclimation, which suggests that the plant cold stress response is a complex, vital phenomenon that involves more than one pathway. The C-Repeat Binding Factor (CBF) pathway is the most important and well-studied cold regulatory pathway that imparts freezing tolerance to plants. The regulation of freezing tolerance involves the action of phytochromes, which play an important role in light-mediated signalling to activate cold-induced gene expression through the CBF pathway. Under normal temperature conditions, CBF expression is regulated by the circadian clock through the action of a central oscillator and also day length (photoperiod). The phytochrome and phytochrome interacting factor are involved in the repression of the CBF expression under long day (LD) conditions. Apart from the CBF regulon, a novel pathway involving the Z-box element also mediates the cold acclimation response in a light-dependent manner. This review provides insights into the progress of cold acclimation in relation to light quality, circadian regulation, and photoperiodic regulation and also explains the underlying molecular mechanisms of cold acclimation for introducing the engineering of economically important, cold-tolerant plants.

## 1. Introduction

Plants are continuously exposed to several abiotic stresses, such as drought, salinity, and cold, and have thus developed various protective mechanisms against these stressors. Among these abiotic stresses, low temperature (LT) is a very important factor, as LT severely affects plant growth and development, which control crop distribution and yield. Exposing plants to LTs in the range of 0–12 °C produces chilling injury, while temperatures below 0 °C result in freezing injury; these processes are collectively known as cold stress [1]. Most temperate plants have the ability to acquire chilling and freezing tolerance after being exposed to mildly low but nonfreezing temperatures, a process known as cold acclimation [2–4]. Several studies using the model plant *Arabidopsis* have increased our understanding of cold acclimation. These studies, which have been reviewed extensively, suggest that cold acclimation is a complex process accompanied by metabolic reprogramming and transcriptional responses [5,6]. The C-Repeat Binding Factor (CBF) transcriptional pathway plays an important role in cold acclimation; this pathway can activate *COR* (COLD RESPONSIVE) genes [7]. In addition to temperature, light also acts as an external signal that affects plant growth. Rather than acting independently, the proper integration of both cues results in the increased fitness of plants to survive changing environments. Various studies have shown that light is necessary for cold acclimation in plants, specifically for increasing freezing tolerance [8,9]. Previously, it has been reported that light positively regulates cold-induced gene expression, including CBFs [10–12], which require phytochromes [13]. Apart from the CBF regulon, light mediates cold acclimation through a novel low temperature response element (LTRE) referred to as the Z-box element. The activation of LTRE-dependent, cold-induced genes requires functional HY5 (ELONGATED HYPOCOTYL 5), a well-known positive regulator of light-dependent signal transduction that is controlled post-translationally by COP1 (CONSTITUTIVE PHOTOMORPHHOGENIC 1) [14].

The role of the circadian clock in freezing tolerance has been previously documented. The induction of *CBF* genes is controlled by central oscillators of the clock, namely CCA1 (CIRCADIAN CLOCK ASSOCIATED-1) and LHY (LATE ELONGATED HYPOCOTYL), which are essential for increasing CBF expression to impart maximum cold tolerance to plants at both non-acclimating and cold-acclimating temperatures [15,16]. Photoperiod is a good indicator of seasonal changes in plants and regulates the CBF induction pathway. Most woody plants have the ability to sense the shortening of day length at the beginning of winter, which alerts plants to prepare for upcoming cold stress [17,18]. The roles of phytochrome B (*PHYB*) and phytochrome interacting factors (*PIF4* and *PIF7*) involve the photoperiodic regulation of CBF expression, as demonstrated in the model plant *Arabido*psis [19]. Recent advances in identifying cold-inducible genes offer considerable potential for crop improvement. Various *CBF* genes have been isolated and incorporated into different plant species to enhance freezing tolerance at LTs.

## 2. Cold Acclimation and the *CBF* Regulon

Plants from tropical, sub-tropical, and temperate regions have different responses to cold stress due to differences in their abilities to undergo cold acclimation; plants from temperate environments, for example, are more tolerant of freezing conditions than tropical plants [4,20]. Cold acclimation involves extensive physiological and biochemical changes required for the stability of subcellular structures, which are essential for membranes and cytoskeletons [21,22]. In addition, during cold acclimation, pigments accumulate to protect plants from photoxidative stress. Moreover, low compatible solutes accumulate to maintain the osmotic balance, and cryoprotective proteins and antifreeze proteins accumulate to reduce cellular damage [4]. In *Arabidopsis*, approximately 4%–20% of the genome is regulated by cold stress. Moreover, some of these genes are also regulated by other abiotic stresses, such as dehydration and high salt, indicating that these processes share a common signaling pathway [23,24]. Thus, understanding the molecular mechanisms underlying cold acclimation will be helpful for introducing cold tolerance along with increase in tolerance level to other abiotic stresses, such as drought and salinity into economically important plants. In *Arabidopsis* the fact that 12% of the cold-regulated genes are controlled by CBFs, which represent 4% of the total number of *Arabidopsis* genes [25,26], highlights the importance of *CBF* genes in the cold stress response of plants.

The CBF pathway is a well-studied cold regulatory pathway that plays an important role in freezing tolerance in *Arabidopsis*. CBFs, which are also known as *Dehydration-Responsive Element Binding Factors* (*DREBs*), comprise a small family of three transcriptional activators [7,27,28]. Promoter analysis of cold-inducible genes revealed a 9-bp DNA element known as a dehydration-responsive element (DRE; TACCGACAT), which induces gene expression in response to LT and dehydration but not to ABA, whereas a 5-bp core DNA sequence from DRE known as C-repeat (CRT; CCGAC) is sufficient to activate gene expression under cold stress [29,30]. *CBF1*, *CBF2*, and *CBF3* encode closely related members of the AP2/ERF family of DNA-binding proteins, which recognize the promoters of cold-inducible genes containing CRT/DRE sequences and can regulate the transcriptional response. CBF proteins are characterized by the presence of two signature sequences, PKKP/RAGRxxKFxETRHP (abbreviated PKKPAGR) and DSAWR, located immediately upstream and downstream of the AP2 domain, respectively. The signature sequence PKKPAGR is important for CBF1 in the recognition of the *cis*-element [31,32]. The *CBF1*, *CBF2*, and *CBF3* genes are located in tandem on chromosome IV and lack introns. Apart from cold acclimation response, the constitutive CBF expression during early stages of *Arabidopsis* development imparts tolerance to fragile plants against unexpected low temperatures and related stresses [33]. Under normal temperatures the expression of CBFs showed circadian rhythm and was also influenced by the day length (photoperiod) for example, it is repressed under long day condition as against short day [11,16,19,34]. Despite the absence of the CRT-CCGAC sequence in the promoter region, the expression of *CBF* genes is induced specifically in response to LT but not in response to dehydration or high salt [28]. The expression of *CBF* genes was found to be dependent on a MYC-type basic helix-loop-helix transcription factor *ICE1* (INDUCER Of CBF EXPRESSION 1), which can bind to a MYC recognition element in the *CBF3* promoter [35]. ICE1 is constitutively expressed-nuclear localized protein, which induces the expression of CBFs in response to cold stress, and this activation depends on posttranslational modifications like phosphorylation and sumoylation [35,36]. A RING finger ubiquitin E3 ligase, *HOS1* (HIGH EXPRESSION OF OSMOTICALLY RESPONSIVE GENE 1) regulates ICE1 activity by the ubiquitination/26S proteosome pathway [37,38]. However, it has been speculated that CBFs exhibit differential regulation during cold acclimation. The expression of *CBF1* and *CBF3* is followed by the expression of *CBF2*, which acts as a negative regulator of *CBF1* and *CBF3*; this regulation is necessary for optimal activity of the CBF regulon [33,39]. On the contrary, CBF3 might be involved in negative regulation of *CBF2* expression as evidenced from the increased level of *CBF2* in *ice1* mutant accompanied by reduced levels of *CBF3* [35]. Recently, *HOS1* was found to be negative regulator of *CO* (CONSTANS) abundance in response to photoperiod, and controls flowering. It has also been suggested that *HOS1* might function as an integrative link between photoperiodic regulation of CO level and *CBF* expression through ICE1 degradation [40]. Thus, the current review focuses on the contribution of light quality, circadian clock and photoperiod on the expression pattern of *CBF1*, *CBF2* and *CBF3* genes under cold stress response. The list of genes involved in these pathways is described in Table 1.

## 3. Light Quality and Cold Acclimation

Light is an important factor for plants, as it provides an informational signal and a source of energy for plants [44]. Plants are equipped with special mechanisms to sense several parameters of ambient light signals, including light quality (wavelength), quantity (fluence), duration (including daylight), and direction. In *Arabidopsis*, light is absorbed by different photoreceptors. Phytochromes (PHYA, PHYB, PHYC, PHYD, and PHYE) mainly absorb red (R) and far-red (FR) light, while cryptochromes (CRY1, CRY2, and CRY3) and phototropins (PHOT1 and PHOT2) absorb blue and UV-A light, respectively [45–47]. Phytochromes exist in two forms, *i.e.*, the inactive Pr form and the active Pfr form. After absorbing a photon, the inactive red-light-absorbing Pr form is photoconverted into the physiologically active far-red-absorbing Pfr form, which, in turn, is transformed back into the Pr form upon absorption of FR light. PHYs regulate the expression of several downstream genes [46].

Light is required for the expression of several cold-regulated genes (*CORs*) in different plant species. Phytochromes play an important role in light-mediated signaling to activate cold-induced gene expression through the CBF pathway [10,11]. The accumulation of *COR14b* in etiolated barley plants is mediated by red or blue light but not by FR or green light pulses, indicating the involvement of phytochromes and blue light receptors in the control of *COR14b* gene expression [10]. During twilight (dusk and dawn), the decrease in the R/FR ratio results in the conversion of active Pfr to the inactive Pr form [48]. The decrease in the R/FR ratio can result in the increased expression of CBFs, which in turn increases the expression of *COR* genes in *Arabidopsis*. Similarly, *phyB* and *phyD* mutants show an increase in *COR15a* expression under a low R/FR ratio at 16 °C, which indicates that these phytochromes play a negative role in regulating the expression of CBF target genes under high R/FR light conditions [34]. Several different studies concerning how red and FR light conditions are related to cold acclimation have been reported. For instance, when the woody plants *Cornus* and *Weigela* are exposed to a brief pulse of red light during a dark period, cold acclimation is suppressed, but this suppression is relieved if the red light pulse is followed by FR light [49]. Also, in another study, cold acclimation was found to be promoted in red osier dogwood (*Cornus stolonifera*) if the plants were exposed to FR light by the end of the day [50]. Thus, light quality is a crucial factor involved in the cold acclimation response.

Moreover, cold-treated, etiolated *Arabidopsis* seedlings exhibit greater *COR15a* transcript accumulation in the light than in the dark, suggesting that the CBF pathway is tightly linked to light signaling [11]. Also, the expression of several cold-responsive genes is more strongly regulated under cold/light conditions than under cold/dark condition, as demonstrated by microarray analysis [12]. Apart from cold/light and cold/dark, the different light intensity was found to be inducing different biochemical changes responsible for enhancing freezing tolerance which might be dependent on the modulation of photosystem II (PSII) excitation pressure [51–53]. But exact mechanisms of the contribution of light quantity in CBFs expression during frost hardening to enhance the freezing tolerance are still unknown.

HY5, which is a key regulator of photomorphogenesis, is regulated by the E3 ubiquitin ligase COP1, a crucial repressor of light signaling. In the light, COP1 is excluded from the nucleus, allowing HY5 stabilization and activation of light-responsive genes [54]. Moreover, COP1 is depleted from the nucleus in response to cytokinins and gibberellins [55]. However, in response to UV-B, COP1 is not excluded from the nucleus and is required for *HY5* gene activation [56]. Recently, approximately 10% of all cold-inducible genes of *Arabidopsis* that are involved in cold acclimation were shown to be regulated by the HY5 pathway. This newly identified pathway involves *HY5*, *COP1*, the Z-box element, and a motif involved in light-regulated gene expression [57]. The transcript level of *HY5* is regulated by LT through a CBF- and ABA-independent pathway, and the HY5 protein is regulated post-translationally by the nuclear depletion of COP1 [14]. HY5 is upregulated at LTs and mediates the induction of several cold-inducible genes through the Z-box, which constitutes a LTRE. These cold-inducible genes include *CHS* (Chalcone Synthase, *CHI* (Chalcone Isomerase) and *FLS* (Flavonal Synthase), which are involved in anthocyanin biosynthesis [58]. The accumulation of anthocyanin protects the photosystems of plants from ROS accumulation during abiotic stress, including LT stress [59]. Thus, the above results help elucidate the complex molecular mechanisms integrating LT and light signals to impart cold acclimation to plants.

## 4. Circadian Regulation of Cold Acclimation

The circadian clock is an endogenous mechanism by which most organisms synchronize their physiological and metabolic processes with diurnal and seasonal changes [60]. The clock generates biological rhythms with a period of approximately 24 h that can integrate multiple external signals such as light, temperature, and nutrient availability [61]. Every aspect of plant growth and development is regulated by the circadian clock, resulting in enhanced fitness and survival [62–65]. The circadian clock of *Arabidopsis* consists of three interlocking regulatory feedback loops, namely the core feedback loop, the morning loop, and the evening loop [61,66,67]. The core feedback loop is composed of CIRCADIAN CLOCK-ASSOCIATED 1 (CCA1) and LATE ELONGATED HYPOCOTYL (LHY), Myb transcription factors that have partially overlapping functions [68–71], and TIMING OF CAB 1 (TOC1), a PSEUDO RESPONSE REGULATOR (PRR) protein [72]. The increased levels of *CCA1* and *LHY* expression just after dawn repress the expression of *TOC1*, as these elements directly bind to the Evening Element (EE) present in the promoter of *TOC1* [65,73]. The expression level of *TOC1* peaks in the evening, which is important for the induction of both *CCA1* and *LHY* [68]. The repression activity of the TCP transcription factor CCA1 HIKING EXPEDITION 1 (CHE1) toward *CCA1* is inhibited by TOC1 [74], but the mechanism by which *LHY* expression is activated by TOC1 remains unknown. The morning loop is composed of *PRR7* and *PRR9*, which are activated by CCA1 and LHY directly, while PRR7 and PRR9 act as a negative regulator for *CCA1* and *LHY* expression [75,76]. The complex circadian regulatory networks, which are regulated in distinct ways, including transcriptional, post-transcriptional, and posttranslational mechanisms, have recently been thoroughly reviewed [61,77].

Apart from light signals, temperature can also act as an important cue for controlling circadian rhythms. The property of circadian clocks to maintain stable rhythms over a wide range of physiological temperatures is known as “temperature compensation” [78]. The rhythmic expression of core clock genes such as *CCA1*, *TOC1*, *LHY*, and *PRR7* in response to temperature entrainment can be altered after short or long periods of cold exposure [61,79]. Moreover, the loss of function alleles of each of the *PRRs*, *LHY*, *ZTL* (ZEITLUPE), *CCA1*, and *TOC1* genes confer circadian defects after temperature entrainment, which is similar to that of light entrainment, which confers the critical role for these components under a temperature-sensitive circadian system [79]. Recently, increasing evidence has suggested that the circadian clock is involved in freezing tolerance through CBF-dependent and CBF-independent pathways [61]. Under warm temperature conditions, the transcript levels of *CBF1*, *CBF2*, and *CBF3* oscillate, with a peak occurring at approximately 8 h after dawn (Zeitgeber time 8; ZT8) and a trough occurring at approximately ZT20. When plants are exposed to LTs at ZT4, the increase in transcripts level is much greater than that in plants exposed to LTs at ZT16. Similar results were also observed for the cold-responsive transcription factor genes *RAV1* and *ZAT12* [16]. The reason behind these gating effects is that after a short initial cold response, under diurnal conditions, cold reduces the amplitude of cycles for clock components and dampens or disrupts the cycles of output genes, while under continuous light, all cycles become arrhythmic [80]. Moreover, constitutive expression of *CBF* genes in the *prr5/prr7/prr9* triple mutant of *Arabidopsis* produces high levels of cold tolerance. Also, *PRR5/PRR7/PRR9* may act as a negative regulator of the CBF pathway [41]. The bHLH transcription factor PIF7 (Phytochrome Interacting Factor 7) can bind to the G-box present in the promoters of *CBF* genes and act as a repressor of *CBF1* and *CBF2* expression under circadian control [81]. The gating effects on *CBF* gene expression are abolished in CCA1-overexpressing plants, which is consistent with clock control of cold acclimation. The disruption of the circadian clock by cold stress is also observed in some woody plants, such as chestnut [82,83]. CCA1 binds directly to the promoters of *CBF* genes to regulate their expression, which in turns induces cold tolerance. CCA1 and LHY may be required for the induction of *CBF* genes, as well as *COR* genes such as *COR15a*, *COR47*, and *COR78*. Thus, *CCA1/LHY*-mediated output from the circadian clock plays a positive role in plant cold tolerance through the regulation of the CBF pathway [15]. Moreover, temperature-associated alternative splicing is an additional mechanism involved in the operation and regulation of the plant circadian clock [84]. The *CCA1* transcription factor gene also undergoes an alternative splicing event to produce two different forms, namely CCA1α and CCA1β, which differ in their DNA-binding activity due to the lack of DNA-binding domains in CCA1β. Thus, CCA1β can form a non-functional dimer with CCA1α or LHY, which have reduced DNA-binding activity. LT inhibits alternative splicing and results in a reduction in CCA1β production, which allows CCA1α to positively regulate *CBF* gene expression [42].

## 5. Photoperiodic Regulation of Cold Acclimation

Generally speaking, it is still not clear how trees and shrubs in temperate climates survive the winter to produce leaves and bloom each spring. The ability of these plants to survive is based on an adaptive mechanism where a plant enters into a state of dormancy and develops freezing tolerance. The ability of woody plants to tolerate extreme freezing temperatures is achieved by sequential stages of cold acclimation. The first stage is initiated by short day length (SD), and the next stages are initiated by LTs and freezing temperatures, respectively [18]. With the onset of early autumn, plants sense the shortening day length and initiate developmental programs that result in the cessation of growth and an increase in freezing tolerance. As the season progresses and the temperatures become colder, plants sense the LTs and respond by increasing their freezing tolerance [85,86]. To sense seasonal changes, a phenomenon known as photoperiodism becomes well-established in plants; this phenomenon is seasonally controlled by environmental signals [87].

The proper integration of both LT and short photoperiods has a direct impact on plants at the physiological, metabolic, and transcriptional levels. For instance, the combination of these two environmental cues has an additive effect on cold acclimation, leading to an increase in freezing tolerance in woody plants, such as silver birch (*Betula pendula* Roth) [88]. Similarly, in the herbaceous plants different climatic ecotypes exhibit different levels of freezing tolerance in response to photoperiod. In *Arabidopsis*, *Landsberg erecta* exhibits greater freezing tolerance than the sub-tropical *Cape Verde Islands* variety. These varieties differ in their freezing tolerance before and after cold acclimation, as well as in their cold acclimation response in relation to photoperiod conditions. The genetic basis of this variation is explained by using the QTL mapping approach, where QTLs were mapped under LD and SD photoperiods [89]. The similar results has been observed in barley and wheat varieties, where the highly sensitive SD varieties delayed the vegetative/reproductive transition under SD conditions to increase the low temperature tolerance and the less sensitive SD varieties shows similar respond to low temperatures during both SD and LD treatments [90].

The role of phytochrome has been speculated on in photoperiodic responses [13]. The overexpression of oat phytochrome A in hybrid aspen prevents cold acclimation in response to SD and significantly changes the critical photoperiod which indicates *PHYA* may be involved in the detection of photoperiod in a temperate-zone deciduous tree. Moreover, SD and LT induce cold acclimation independently [91]. The presence of photoperiod-insensitive woody species of the Rosaceae family, such as apple and pear, clearly indicate the role of photoperiod in cold acclimation, as only LT, not photoperiod, can influence the cold acclimation of these plants [92]. Although considerable progress has been made in understanding cold acclimation, little is known about the molecular mechanisms underlying how plants perceive LT and SD conditions to acclimate to cold conditions. Recently, it has been reported that freezing tolerance in response to SD conditions (versus LD conditions) increases in *Arabidopsis*. This process involves photoperiodic regulation of the CBF pathway. In warm-grown plants under LD conditions, the CBF pathway is repressed by the two bHLH transcription factors, *PIF4* and *PIF7*, which bind directly to the G-box present in the promoters of *CBF* genes. PIF4 and PIF7 proteins accumulate under LD conditions, but their level decreases under SD conditions to release the repression, resulting in an increase in freezing tolerance. This photoperiodic regulation requires functional phytochrome B (*PHYB*). The differential expression levels and stability of *PIF4* and *PIF7* under LD and SD conditions may be regulated by *PHYB* to control *CBF* gene expression-mediated cold acclimation. The CBF region is controlled by the circadian clock, and the same holds true for PIF transcription factors. Thus, it is possible that output from the circadian clock can integrate the photoperiodic regulatory pathways [19].

## 6. Genetically Engineering Cold-Stress Tolerance in Crop Plants

Cold stress adversely affects plant growth and development, which results in an annual loss of approximately $2 billion worldwide [93]. The developments of molecular genetics and biotechnology techniques have made it possible to generate various genetically modified plants that are tolerant of many environmental stressors, including LT. When plants are exposed to LT, numerous physiological and molecular changes occur to help the plants adapt to the changing environment. As discussed above, the CBF regulon is the most important pathway in the cold-stress response. This regulon is highly conserved in flowering plants irrespective of their freezing tolerance ability. For example, temperate cereals, such as barley, rye, and wheat, as well as cold-sensitive plants, such as tomato, maize, and rice, employ the CBF pathway [94,95]. The differential regulation of the CBF pathway by light quality, photoperiod, and circadian rhythms results in different levels of cold tolerance in plants. Thus, the CBF pathway is an important target for genetic engineering to improve cold tolerance in winter cultivars.

Recently, several studies have shown that freezing tolerance can be increased by overexpressing *CBF* genes in different plant species such as canola (*Brassica napus*) [94] and tomato (*Lycopersicon esculentum*) [96]. Similarly, exogenous expression of CBFs from different crops such as wheat (*TaDREB2* or *TaDREB3*) greatly increases drought and frost tolerance in transgenic wheat and barley [97]. Moreover, overexpression of rice *OsDREB1A* or *OsDREB1B* or barley *HvCBF4* increases cold tolerance in transgenic rice [98,99]. The major drawback of constitutive expression of *CBF* genes is that the expression of these genes can cause growth retardation and reduced yields, which can be minimized by using different promoters or *CBF* genes from other crops [99,100]. Along with CBF, other transcription factors that are also involved in cold acclimation and stress tolerance mechanisms have been employed in the genetic engineering of stress-tolerant plants. Also, some cold-tolerance genes have been identified for use in crop improvement, as has been reviewed recently [93]. As explained above, many cold-inducible genes have been isolated and identified in studies of cold tolerance employing *Arabidopsis* as a model plant. However, while technology exists for engineering freezing tolerance in crops, this technology has been studied in only a limited number of crop species. Even the well-studied CBF regulon can only be applied to some crop species. Therefore, advanced techniques are needed to enable the rapid, accurate incorporation of cold-tolerance genes into different crop plants.

## 7. Conclusions

**Figure 1:**
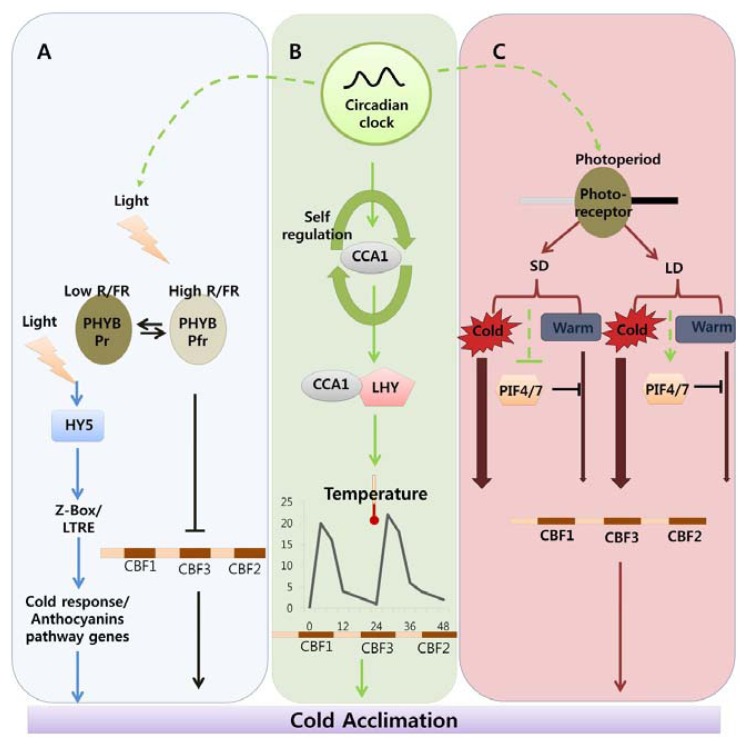
Complex regulatory network of cold acclimation, including key components. (**A**) Light signalling; **(B**) Circadian regulation; (**C**) Photoperiodic regulation. 
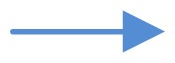
 Indicates cold acclimation pathway in which *HY5* regulates the expression of anthocyanin pathway genes through by Z-box/LTRE to control photo-oxidative stress generated by low temperature. → Indicates cold acclimation pathway in which phytochromes negatively regulates the expression of CBF regulon under high red/far red light condition. 
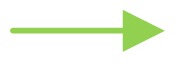
 Indicates circadian clock-regulated cold acclimation pathway in which CCA1 showed selfregulation activity through alternate splicing event in response to cold and it can form heterodimers with LHY to bind directly to promoter region of *CBF* genes. 
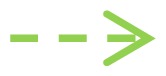
 Indicates influence of circadian clock over light and photoperiod mediated cold acclimation pathway which is unclear. 
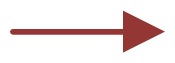
 Indicates photoperiod involvement in cold acclimation pathway which is regulated by the *PHY B* and the activity of *PIF4/7* in response to SD and LD which can repress the CBF expression under LD by direct binding to promoter region. 
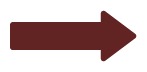
/ 
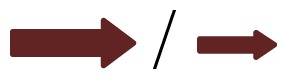
 Width of arrow indicates the expression levels of CBF genes in response to photoperiod. (For detailed explanation please see the text).

Plants rely on light and temperature signals to adapt to changing environmental conditions. The circadian clock is a mechanism that enables plants to synchronize growth according to growing conditions. Due to their sessile nature, plants have evolved complex mechanisms to deal with a variety of stresses, including low-freezing temperature. As mentioned above, as cold stress can lead to a loss of approximately $2 billion per year worldwide, much attention has focused on improving crop yields under LT conditions. Major breakthroughs in the field of cold tolerance have come from the discovery of the CBF regulon. Using a transgenic approach to constitutively overexpress *CBF* genes in crop plants such as canola, wheat, rice, and tomato has been helpful for improving cold tolerance in plants, although these transgenic plants exhibit some unwanted side effects such as retarded growth and reduced yields. Thus, to avoid the negative effects of the exogenous expression of these transgenes, it is important to understand the molecular mechanisms controlling the CBF pathway. There have been recent advances in elucidating cold acclimation in the model plant *Arabidopsis*; a model derived from the results of these experiments is shown in Figure 1. Cold acclimation is a complex mechanism that can be defined as an increase in the freezing tolerance of plants after exposure to low-nonfreezing temperatures. In plants, cold acclimation is mediated by CBF-dependent or CBF-independent pathways, such as ABA-dependent pathways. The role of the CBF regulon, which is at the center of the cold acclimation pathway, has been well-studied in processes such as plant responses to light, circadian clock-controlled processes, and photoperiodic responses. Light plays a positive role in the induction of *CBF* genes and photoreceptors, acts as a repressor of CBF expression which is important because the constitutive expression of CBF leads to growth retardation. Moreover, HY5, a positive regulator of light-dependent signal transduction, is important for the activation of cold acclimation genes through a CBF-independent pathway that includes Z-box element/LTRE as a cis-acting element. The central oscillators of the circadian clock, namely *CCA1* and *LHY*, are important for the induction of *CBF* genes in response to cold stress, as the induction of these genes depends on the time of day at which plants have been exposed to LT. Such circadian regulation is important in nature because lower temperatures tend to coincide with the reduction in light that occurs during the winter season. A temperature-dependent alternate splicing mechanism regulates the activity of CCA1, which is important for DNA binding to the promoter regions of *CBF* genes. The circadian clock can influence the light and temperature-mediated induction of *CBF* genes, as indicated by the dotted line in Figure 1. Photoperiod is critical for the expression of *CBF* genes, and it is mediated by *PHYB* and *PIF4/7*. The transition from summer to winter results in the shortening of day length. Thus, short days act as a good indicator of upcoming cold stress, which acts as a signal for plants to begin the process of cold acclimation. Under LD conditions, PIF4/7 binds to the G-box of *CBF* genes and represses the expression of these genes, while under SD conditions; the PIF4/7 levels are repressed, which results in the induction of *CBF* genes and cold acclimation. In summary, CBF-mediated cold acclimation is regulated by complex networks of the circadian clock and entraining signals such as light and temperature. In the near future, it will be important to produce cold-tolerant crop plants using our current knowledge of the complex process known as the cold acclimation pathway.

## Figures and Tables

**Table 1:** List of genes involved in cold acclimation in plants in relation to light quality, circadian clock and photoperiodism.

Symbol	AGI code	Full name	Mechanism and phenotype	Ref.
**A. Genes involved in light signaling and cold acclimation response pathway**				
*CBF2*	AT4G25470	C-REPEAT/DRE BINDING FACTOR 2	*CBF2* repress the expression of *CBF1/3* and the *cbf2* mutant showed enhanced cold tolerance	[39]
*CBF1*	AT4G25490	C-REPEAT/DRE BINDING FACTOR 1	*CBF1/3* are the positive regulators for cold acclimation and RNAi lines are impaired in cold tolerance	[33]
*CBF3*	AT4G25480	C-REPEAT BINDING FACTOR 3		
*HY5*	AT5G11260	ELONGATED HYPOCOTYL 5	*HY5* positively regulates cold induced gene expression through Z-box/LTRE and hy5 mutant is sensitive	[14]
*PhyB*	AT2G18790	PHYTOCHROME B	repression of the CBF regulon in high R/FR is mediated by *PHYB* and *PHYD* and thus, *phyB* and *phyD* mutants showed increased cold tolerance	[34]
*PhyD*	AT4G16250	PHYTOCHROME D		
**B. Genes involved in circadian clock and cold acclimation response pathway**				
*PRR5/7/9*	AT5G24470/AT5G02810/AT2G46790	PSEUDO RESPONSE REGULATOR 5/7/9	acts as a negative regulators for CBF pathway and thus, triple mutant *prr5/7/9* showed increased cold tolerance	[41]
*CCA1*	AT2G46830	CIRCADIAN CLOCK ASSOCIATED 1	CCA1 binds to promoter of CBFs and promotes cold acclimation which is self- regulated in response to cold stress by alternative splicing mechanism	[15,42]
*LHY*	AT1G01060	LATE ELONGATED HYPOCOTYL	*LHY* is also important along with *CCA1* for circadian regulation of CBFs and double mutant *cca1-11/lhy-21* is impaired in freezing tolerance	[15]
*GI*	AT1G22770	GIGANTEA	*GI* positively regulates freezing tolerance via a CBF-independent pathway and *gi-3* mutant is susceptible to freezing due to impaired sugar metabolism	[43]
**C. Genes involved in photoperiodism and cold acclimation response pathway**				
*PIF4*	AT2G43010	PHYTOCHROME INTERACTING FACTOR 4	*PIF4* and *PIF7* function redundantly and binds to G-box present in the promoter of CBF genes to repress its expression under LD conditions	[19]
*PIF7*	AT5G61270	PHYTOCHROME INTERACTING FACTOR 7		
